# MATLAB-based Methods Allow Precise, High-Throughput Quantification of Nuclear Morphology and Texture in Tachycardiomyopathy

**DOI:** 10.1369/00221554251332331

**Published:** 2025-04-11

**Authors:** Moritz N.S. Mayer, Lisa M. Köhler, Michael Paulus, Sabine Iberl, Maria Heinrich, Stefan Wagner, Lars S. Maier, Alexander Dietl

**Affiliations:** Department of Internal Medicine II, University Hospital Regensburg, Regensburg, Germany; Department of Internal Medicine II, University Hospital Regensburg, Regensburg, Germany; Department of Internal Medicine II, University Hospital Regensburg, Regensburg, Germany; Department of Internal Medicine II, University Hospital Regensburg, Regensburg, Germany; Department of Internal Medicine II, University Hospital Regensburg, Regensburg, Germany; Department of Internal Medicine II, University Hospital Regensburg, Regensburg, Germany; Department of Internal Medicine II, University Hospital Regensburg, Regensburg, Germany; Department of Internal Medicine II, University Hospital Regensburg, Regensburg, Germany

**Keywords:** digital image analysis, hypertrophy, nuclear morphometry, tachycardiomyopathy, texture

## Abstract

The understanding of cardiomyopathies is hindered by a lack of quantitative histologic data. To address this methodical gap, we wrote a MATLAB-based image analysis platform to quantify nuclear and cellular disarray. We validated its utility in an animal model of tachycardiomyopathy (T-CM), whose ultrastructural remodeling processes have only partially been characterized and differ substantially from more prevalent cardiomyopathies. Six rabbits received right ventricular pacemaker implants. Three animals were paced incrementally up to 380 bpm for 30 days to induce T-CM. In three control rabbits, the pacemaker remained inactive (SHAM). Left ventricular tissue was collected, fixed in formalin, embedded in paraffin, stained, and digitized for nuclear morphometry, texture analysis, orientation analysis, and vascular architecture evaluation. Nuclear segmentation performed by the software was highly accurate, closely matching manual counts (mean manual nuclear count per slide = 81.3 ± 3.8, mean automated nuclear count per slide = 81.9 ± 4.3, *r* = 0.981, *p*<0.001). In T-CM, nuclei were enlarged [SHAM (a.u.) = 2362, T-CM (a.u.) = 2660, *p*=0.0042]. Texture patterns differed between the groups with higher nuclear contrast in T-CM [SHAM (a.u.) = 0.0169, T-CM (a.u.) = 0.0247, *p*=0.0149], highlighting structural remodeling at the nuclear level. Median vessel size increased in T-CM [SHAM (a.u.) = 1532, T-CM (a.u.) = 2421, *p*<0.0001]. In conclusion, our MATLAB-based image analysis platform allows high-throughput quantification of nuclear and extracellular disarray. It identified enlargement of nuclei and increased nuclear contrast as part of ultrastructural remodeling in tachycardiomyopathy:

## Introduction

Cardiomyopathies encompass a heterogeneous group of cardiac dysfunctions that vary widely in etiology, diagnosis, treatment, and prognosis. They can be distinguished using electrocardiogram, echocardiogram, cardiac magnetic resonance imaging, and biopsies.

Cardiac histologic samples are primarily analyzed using qualitative approaches,^1^ which, while valuable for characterizing histopathologic features, lack the precision required for quantitative comparability. This gap in quantitative analysis limits our ability to fully understand and discriminate between different types of cardiomyopathies.

Regarding nuclear morphology, alterations in nuclear size,^2^ shape,^3^ and density^4^ have been observed. However, these findings have traditionally relied on manual delineation of nuclear borders—an approach that is labor-intensive, susceptible to observer bias, and constrained to a limited range of nuclear parameters.

Therefore, a fully automated, standardized, and quantitative approach is sought after to account for possible shortcomings from observer-dependent evaluation and selective perception.^5^

Despite the critical role of cardiomyocyte alignment in effective electrical depolarization and myofilament contraction,^6^ cardiac cell orientation in cardiomyopathies has not been quantitatively assessed on a histologic scale. Disruption of this ordered architecture, known as myocardial disarray, is a hallmark of pathophysiological remodeling in heart disease.^7–9^ It has been described at different scales, using methodology ranging from diffusion-tensor magnetic resonance imaging (DT-MRI) for analysis of myocardial fiber distribution^10^ to Serial-Block Face Scanning Electron microscopy for analysis of organization at a subcellular level.^11^ While these methods have advanced our understanding of myocardial disarray, most analyses remain qualitative or dependent on subjective assessments. Quantitative methods for cellular orientation, such as the orientational order parameter (*f2D*) introduced by Umeno et al. in 2001,^12^ have shown promise in cell culture studies and experimental cardiology^13^ but have not been broadly applied to cardiac histopathology.

Tachycardiomyopathy, a condition induced by sustained high ventricular rates, leads to heart failure characterized by partial or full recovery of systolic function after the resolution of tachycardia.^14,15^

While metabolic and mitochondrial remodeling in tachycardiomyopathy differs from other heart failure etiologies,^16^ the lack of quantitative microscopic image analysis platforms hinders the identification of histologic markers for early diagnosis. This limits the potential for novel insights into the disease’s unique pathophysiology.

A high-throughput image analysis platform provides a method to close this scientific gap. Moreover, an image analysis platform might also serve a clinical purpose in the future, as diagnosis of tachycardiomyopathy can currently only be confirmed after left ventricular recovery following the elimination of tachycardia.^17^ However, there is a clinical need for early diagnosis. It would be particularly helpful to solve the frequent chicken and egg dilemma of coincident atrial fibrillation and heart failure.^18^ It is often unclear whether atrial fibrillation or heart failure came first, what to treat first, and whether systolic dysfunction is likely to recover fast after rate control.

In the most severe cases of tachycardiomyopathy-induced cardiogenic shock, clear and certain diagnosis is key to plan further therapy and to strengthen a reasonable estimation of prognosis. In intensive care therapy, bridging therapies depend on the likelihood of fast systolic recovery.^19^ Early diagnosis of tachycardiomyopathy renders fast resolution of shock likely. Together, there is a clinical need for early diagnosis, which would also justify taking biopsies in particular cases.

To address these challenges, we present a novel MATLAB-based image analysis platform designed for large-scale quantitative assessment of immunofluorescence-stained cardiomyocytes. This platform introduces quantitative parameters for evaluating cellular disarray, validates their applicability, and utilizes them to analyze nuclear and extracellular remodeling in a rabbit model of tachycardiomyopathy.

## Materials and Methods

### Animal Model

**Figure 1.**
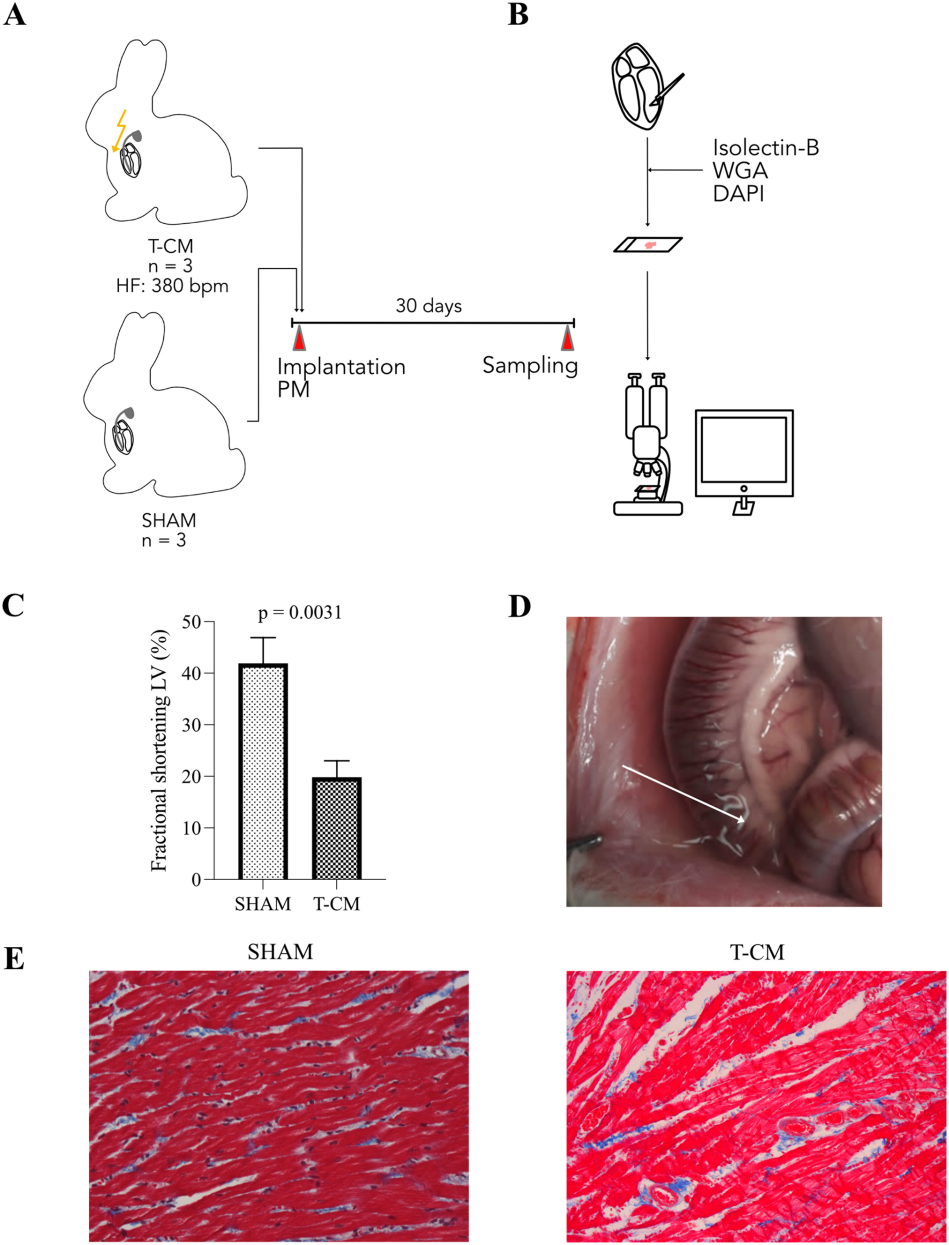
Tachypacing induced systolic heart failure in rabbits. (A) Permanent pacemakers were implanted in rabbits. (B) Left ventricular specimens were stained with DAPI, WGA, and Isolectin B4. (C) Left ventricular fractional shortening declined in T-CM animals. (D) Exemplary depiction of ascites in T-CM animal. (E) Representative images of Masson’s trichrome staining in SHAM (left) and T-CM (right) animals (40× magnification). Abbreviations: T-CM, tachycardiomyopathy; PM, pacemaker; HF, heart frequency; LV, left ventricle; DAPI, 4′,6-diamidino-2-phenylindole.

Tachycardiomyopathy (T-CM) was induced in rabbits using previously established protocols.^16,20^ Six rabbits were surgically implanted with right ventricular pacing devices (Advisa DR MRI SureScan, Medtronic, Minneapolis, MN). T-CM rabbits underwent incremental tachypacing, reaching up to 380 bpm over a 30-day period (Fig. 1). Pacemakers in the control group (SHAM) were not activated. Rabbits were monitored daily to assess overall health, including respiratory rate, body weight, and behavior. The animal study was approved by the institutional and governmental animal care committee (Ref. no. 55.2-2532-2-1121, Regierung von Unterfranken, Germany; University of Regensburg, Germany).

### Transthoracic Echocardiography

Standardized transthoracic echocardiography was performed as previously described.^16,20,21^ Transthoracic echocardiography was used to assess cardiac morphology and function at baseline and on days 10, 20, and 30. Pacing was temporarily suspended during echocardiographic assessment. A HP Sonos 5500 equipped with a 12 MHz transducer (Philips Healthcare, Amsterdam, The Netherlands) was used. Echocardiography was performed with rabbits in a supine position from the left parasternal view. Left ventricular end-diastolic diameter (LVEDD) and left ventricular end-systolic diameter (LVESD) were measured by two-dimensionally guided M-Mode in the parasternal long axis. Systolic function was determined by calculating fractional shortening (FS) as FS = (LVEDD-LVESD)/LVEDD.

### Tissue Fixation and Staining

The tissue samples for further analyses were removed from the anterior left ventricular wall, cutting a 1 × 1 cm large, transmural sample, left of the left anterior descending artery (LAD) and distal the first diagonal branch. The model used in this study is not a working heart model or a perfused heart model. We did not stop the heart in diastole and the heart was not perfusion fixed.

For fluorescence microscopy, left ventricular tissue was fixed in 4% formaldehyde solution (#F25, Waldeck GmbH & Co KG, Münster, Germany) for at least 24 hr and embedded in paraffin according to standard procedures. Thin 4 µm sections were cut with a microtome and stained with a Wheat Germ Agglutinin (WGA) Alexa Fluor 594 Conjugate (W11262, Thermo Fisher Scientific, Waltham, MA) for visualization of connective tissue. To display vessels/endothelial cells, biotinylated Isolectin B4 (B1205, Vector Laboratories, Newark, CA) and Streptavidin-Alexa Flour 488 (016-540-084, Jackson Immuno Research, West Grove, PA) were used. DAPI was used for nuclear staining (4′,6-diamidino-2-phenylindole, D1306, Thermo Fisher Scientific, Waltham, MA).

### Image Acquisition and Processing

A standardized approach was employed for image acquisition, ensuring microscope settings were optimized according to the unique characteristics of each sample. A Zeiss Observer.Z1 microscope (Carl Zeiss AG, Oberkochen, Germany) was used to capture images at 40× magnification. The imaging process employed three specific filter sets: 49 DAPI, 44 FITC, and 71 HcRed. The software used for image acquisition was AxioVision (AxioVs40x64 4.9.1.0). To ensure consistency, regions containing transversely sectioned cardiomyocytes were specifically selected within the tissue.

All image processing was conducted in MATLAB (MathWorks, Natick, MA) R2021b. The images, stored in tagged image file format (.tiff), were separated into their red, green, and blue (RGB) channels. Before analysis, image matrices underwent contrast-limited adaptive histogram equalization to enhance contrast and optimize image quality.

### Segmentation of Nuclei and Morphologic Evaluation

**Figure 2.**
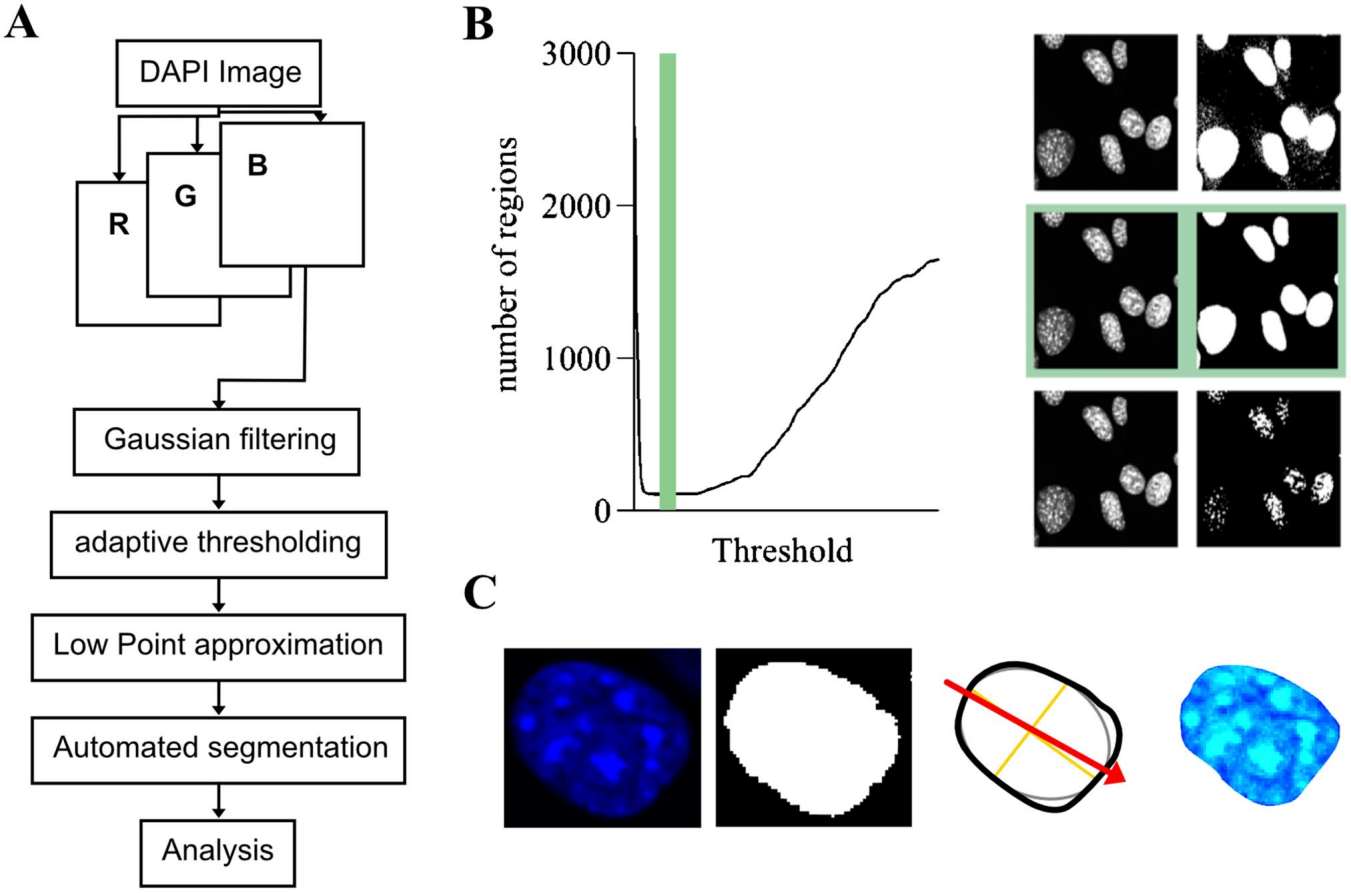
Image analysis workflow. (A) Image segmentation workflow. (B) Illustration of segmentation using adaptive thresholding with low point approximation. (C) Illustration of stages of analysis from left to right: DAPI nucleus, segmented nucleus, analysis of nuclear orientation and shape, selective analysis of nuclear texture. Abbreviations: DAPI, 4′,6-diamidino-2-phenylindole; RGB, Red, Green, Blue.

For the segmentation of DAPI-stained nuclei, the blue channel of the RGB image matrix was selected for further processing. The image was smoothed using a Gaussian filter to reduce noise. Due to light inhomogeneity, an adaptive thresholding technique was applied. This approach was chosen because, at lower thresholds, noise artificially increases the number of segmented regions. As the threshold increases, the influence of noise diminishes, leading to a reduction in the number of identified regions. However, further increases in the threshold result in the disconnection of nuclei, dividing them into regions of euchromatin and heterochromatin, which again increases the number of segmented regions (Fig. 2B). Nuclear shape and size were quantitatively evaluated using mean nuclear size, standard deviation of nuclear size, and eccentricity. The eccentricity was defined as the ratio of the minor and major axis length of an ellipse fitted onto the segmented nucleus. A value close to 0 indicates a circular shape and a value close to 1 indicates an eccentric shape.

### Evaluation of Nuclear Orientation and Its Heterogeneity

**Appendix Figure 1.**
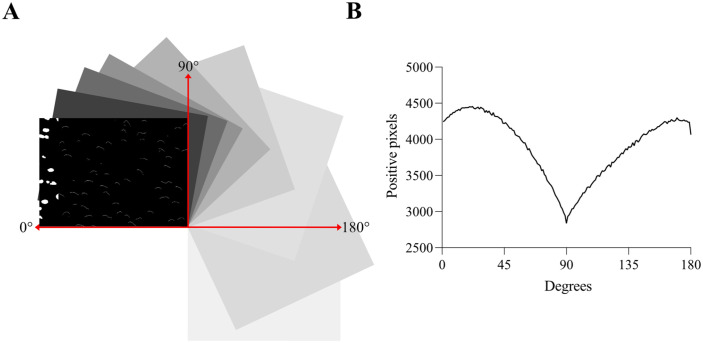
Illustration of rotation histogram. (A) Differential image (foreground right) and small band of segmented nuclear image (foreground left). (B) Resulting rotation histogram.

After successful nuclear segmentation, nuclear orientation was determined using the function regionprops(). From this, an orientation distribution histogram was generated, and histogram-derived parameters—including mean, standard deviation, kurtosis, and skewness—were collected. Furthermore, the Orientational Order Parameter (OOP, *f2D*) of the segmented nuclear image was calculated as described in Umeno et al.^12^:

$$f_{2D}=2((cos^{2}\left(\theta -\mu_{0}\right))-\frac{1}{2})$$

where θ is the orientation of the considered cell and μ_0_ is the mean orientation of all evaluated cells of the image. The term (cos^2^ $\left(\theta -\mu_{0}\right)$ ) describes the mean value of the entirety of evaluated cells. As the OOP obviates the pattern of orientation distribution in cellular clusters, we evaluated the co-occurrence of orientation of neighboring nuclei using a parameter we named orientation co-occurrence parameter (OCP) and defined it as follows:

$$OCP=\frac{1}{n}\overset{n}{\underset{i=1}{\sum }}\cos(\theta_{n}-\theta_{n+i})$$

where $\theta_{n}$ is the orientation of the analyzed nucleus and $\theta_{n+i}$ is the orientation of the neighboring nucleus in a defined direction (90°) and *n* is the number of segmented nuclei. Furthermore, a second method was used to evaluate nuclear orientation. Here, a differential image was created from the segmented image to delineate sudden changes in pixel intensity (nuclear borders) from one direction. This process was repeated for 180 times from the angles 0° to 180° with an increment of 1°. For each direction, the number of pixels belonging to nuclear edges was counted and a histogram was derived containing the number of positive pixels for each degree (0°–180°). From this rotation histogram (Appendix Fig. 1), standard histogram parameters such as mean, standard deviation, kurtosis, and skewness were calculated.

### Evaluation of Nuclear Texture

To selectively assess nuclear texture, a nuclear mask was created, retaining only pixel intensity values from the primary image corresponding to nuclear regions. From this image, a synthetic image was created only containing nuclear texture. A gray-level co-occurrence matrix (GLCM) was then constructed, and Haralick’s texture descriptors—*contrast*, *correlation*, *entropy*, and *homogeneity*—were computed using the function graycoprops().

### Selective Nuclear Analysis of Cardiomyocytes

Selective extraction of nuclear features of cardiomyocytes as opposed to other nuclei such as endothelial nuclei was implemented using a distance transform matrix from the vessel image. Nuclei centered in between vessels were deemed cardiomyocyte nuclei. Nuclei in close proximity to vessel were excluded.

### Evaluation of Extracellular Orientation

Evaluation of the extracellular orientation was performed on the red channel of the acquired RGB image. As the WGA staining of cardiomyocytes and extracellular matrix span a network occupying the whole image, an object-centric orientation evaluation was not performed. The staining was segmented using an adaptive thresholding algorithm. Subsequently, a rotation histogram (Appendix Fig. 1) was created and histogram parameters such as mean, standard deviation, kurtosis, and skewness were calculated from it.

### Evaluation of Extracellular Texture

To evaluate morphologic properties of the extracellular structures contained in the image quantitatively, an image mask only containing regions of WGA staining was created using adaptive thresholding. From this mask, a gray-level co-occurrence matrix was created and Haralick’s texture descriptors *contrast*, *correlation*, *entropy*, and *homogeneity* were calculated using the function graycoprops(). Values for thickness were drawn through calculation of a distance transform matrix from the image mask.

### Evaluation of Vessel Density

To assess vessel density, images corresponding to vessel staining were segmented. The total area occupied by vessels was measured as well as their number and spatial distribution.

### Statistical Analysis

Data are presented as mean values including standard deviation or 95% confidence interval (95% CI). A two-sided Student’s *t*-test was performed for group comparisons of normally distributed quantities. For all cases in which the *t*-test’s assumptions were not met, a non-parametric unpaired Mann–Whitney test was performed. The strength of association between quantitative measures was assessed by Pearson’s (*r*, normally distributed data) correlation coefficient and Spearman’s correlation coefficient for the cases Pearson’s criteria were not met. In case of a normal distribution, we report the mean and standard deviation (Mean ± SD). For all non-normally distributed data, results are presented as the median along with the 25th and 75th percentiles [Median (25P.: 25th percentile, 75P.: 75th percentile)]. Statistical analysis was performed in GraphPad Prism (version 7 and 8, GraphPad Software Inc., San Diego, CA). For all statistical tests, a two-sided *p*-value <0.05 was considered statistically significant.

### Validation

To validate the newly introduced OCP, we utilized synthetic phantom images with predefined nuclear orientations (Appendix Fig. 3). In addition, manual validation for all automatically calculated parameters was performed through manual delineation of nuclear shape, orientation, and membrane on randomly selected images.

**Appendix Figure 2.**
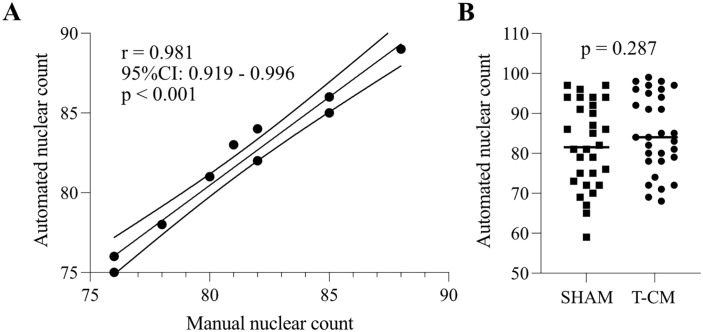
Validation of nuclear count. (A) Manually counted nuclei versus automatically counted nuclei. (B) Nuclear counts per slide in T-CM and SHAM specimens. Abbreviation: T-CM, tachycardiomyopathy.

Automated nuclear counts closely matched manual counts, demonstrating high concordance (Mean manual nuclear count per slide = 81.3 ± 3.8, mean automated nuclear count per slide = 81.9 ± 4.3, *r* = 0.981, *p*<0.001, Appendix Fig. 2A). Automated segmentation also showed strong agreement with manual delineation. Furthermore, T-CM and SHAM did not differ in number of nuclei per slide (Mean number of nuclei per slide T-CM = 84.9, Mean number of nuclei per slide SHAM = 81.9, *p*=0.287, Appendix Fig. 2B).

**Appendix Figure 3.**
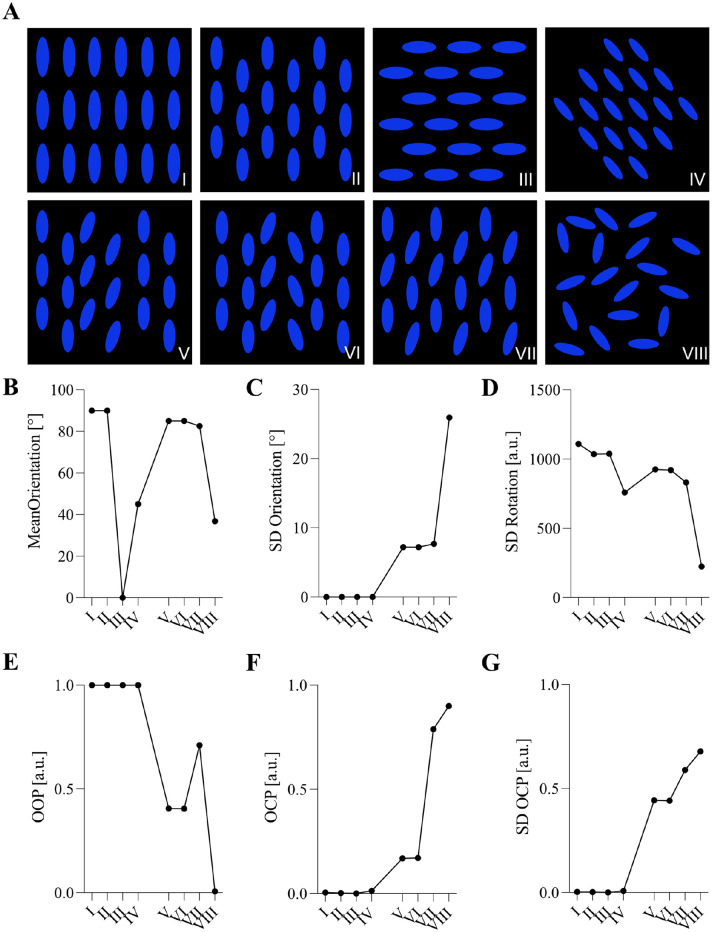
Properties of orientation parameters evaluated on Phantom images. (A) Phantom images I–VIII. (B) Values for mean orientation in degrees of all image regions of the corresponding Phantom image (I–VIII). (C) Values for the standard deviation of the orientation in degrees of all image regions of the corresponding Phantom image (I–VIII). (D) Values for the standard deviation of the positive pixels in rotation (0°–180°) of all image regions of the corresponding Phantom image (I–VIII). (E) Values for the orientation order parameter of all image regions of the corresponding Phantom image (I–VIII). (F) Values for the orientation co-occurrence parameter of all image regions of the corresponding Phantom image (I–VIII). (G) Values for the standard deviation of the orientation co-occurrence parameter of all image regions of the corresponding Phantom image (I–VIII). Abbreviations: SD, standard deviation; OOP, orientation order parameter; OCP: orientation co-occurrence parameter; a.u., arbitrary unit.

To assess the characteristics of OCP and OOP, we analyzed phantom images with predefined orientation properties (Appendix Fig. 3C–G). We could determine that the automatically assumed orientation was in concordance with the manually defined orientation (Appendix Fig. 3B).

## Results

### Tachypacing Induces Systolic Heart Failure

Incremental tachypacing for 30 days induced severe left ventricular dysfunction (Fig. 1C). T-CM rabbits exhibited hallmark signs of advanced heart failure syndrome, including pleural and pericardial effusion as well as ascites (Fig. 1D).

Despite the presence of severe heart failure, Masson’s trichrome staining did not reveal detectable fibrosis (Fig. 1E).

### Nuclear Remodeling in T-CM

**Figure 3.**
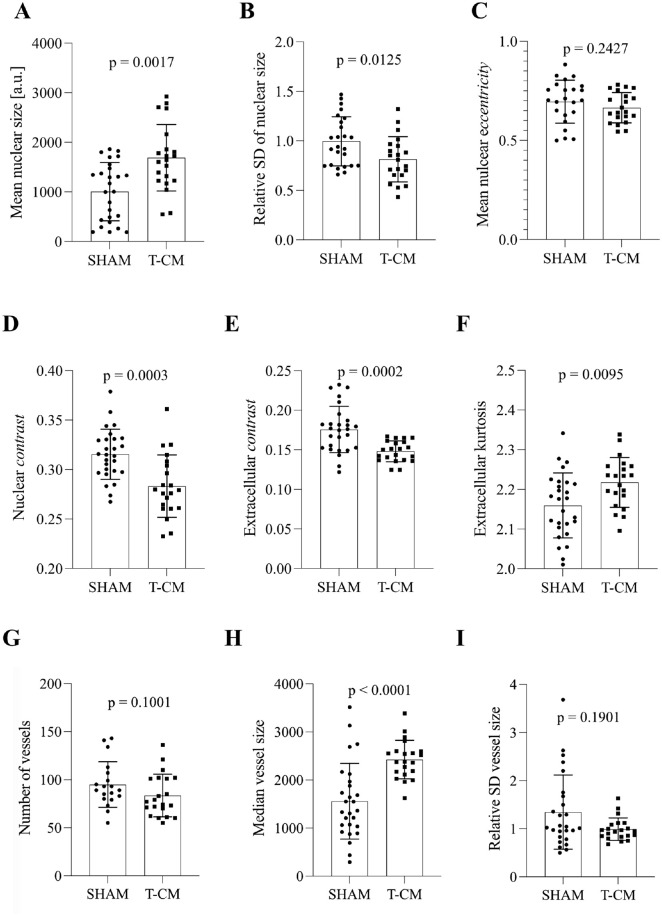
Descriptors of morphology, texture, and vessel architecture in selective cardiomyocyte analysis. (A) Mean nuclear size in DAPI-stained samples from left ventricular tissue of SHAM and T-CM rabbits. (B) Relative standard deviation of nuclear size in DAPI-stained samples from left ventricular tissue of SHAM and T-CM rabbits. (C) Mean nuclear eccentricity in DAPI-stained samples from left ventricular tissue of SHAM and T-CM rabbits. (D) Nuclear contrast in DAPI-stained samples from left ventricular tissue of SHAM and T-CM rabbits. (E) Extracellular contrast in WGA-stained samples from left ventricular tissue of SHAM and T-CM rabbits. (F) Extracellular kurtosis in WGA-stained samples from left ventricular tissue of SHAM and T-CM rabbits. (G) Number of vessels per slide in SHAM and T-CM animals. (H) Median size of vessel [a.u.] in SHAM and T-CM animals. (I) Relative standard deviation of vessel size in SHAM and T-CM animals. Abbreviations: T-CM, tachycardiomyopathy; SD, standard deviation; DAPI, 4′,6-diamidino-2-phenylindole; a.u., arbitrary unit. Mean ± standard deviation.

First, we hypothesized that T-CM entails specific nuclear remodeling. To investigate this, we quantitatively evaluated nuclear size, texture, and orientation. Nuclear sizes in left ventricular specimens differed between SHAM and T-CM animals [Mean SHAM (a.u.) = 2364 ± 295.2, Mean T-CM (a.u.) = 2612 ± 254.6, *p*=0.0042], where the nuclei in T-CM animals were notably larger. This includes nuclei from cardiomyocytes, epithelial nuclei, and nuclei of infiltrating immune cells. Their intrasample variability of size, however, did not differ [Mean SHAM (a.u.) = 1582 ± 276.4, Mean T-CM (a.u.) = 1689 ± 257.8, *p*=0.1637]. Nuclear eccentricity did not differ between the two experimental groups [Mean SHAM (a.u.) = 0.6552 ± 0.0319, Mean T-CM (a.u.) = 0.6567 ± 0.0284, *p*=0.6466]. When nuclei of cardiomyopathy were exclusively analyzed (Fig. 3), they were enlarged in T-CM (Fig. 3A). Nuclei from cardiomyocytes in the T-CM group also showed a slightly more homogeneous size distribution indicated by the relative standard deviation of nuclear size (Fig. 3B). The nuclear eccentricity of cardiomyocytes, however, was not different (Fig. 3C). The staining intensity of DAPI and WGA was similar in SHAM and T-CM animals [DAPI: Median SHAM (a.u.) = 102 (25P.: 89, 75P.: 126), Median T-CM (a.u.) = 113 (25P.: 110, 75P.: 116), *p*=0.1186; WGA: Median SHAM (a.u.) = 149 (25P.: 136, 75P.: 170), Median T-CM (a.u.) = 150 (25P.: 132, 75P.: 172), *p*=0.6615].

In summary, our imaging platform provides a precise tool for quantifying nuclear size across all cells or specifically within cardiac myocytes, as well as measuring nuclear eccentricity. Notably, nuclear size was enlarged in T-CM compared with SHAM.

In addition to nuclear size, we wanted to assess whether more could be learnt on nuclear remodeling in T-CM by quantifying nuclear texture. For this purpose, gray-level co-occurrence matrix (GLCM)-derived parameters were calculated. Selective texture analysis of both nuclear and extracellular staining revealed differences in the uniformity of pixel intensity distribution between left ventricular specimens from SHAM and T-CM animals. These differences were assessed using parameters such as *contrast*, *correlation*, *energy*, and *homogeneity*.

DAPI-stained nuclei exhibited decreased nuclear contrast in T-CM animals upon selective nuclear analysis (Fig. 3D). This difference in nuclear texture was further supported by additional GLCM-derived parameters (Appendix Table 1). Interestingly, in the selective analysis of cardiomycyte nuclei, nuclei from T-CM animals showed increased contrast [Median SHAM (a.u.) = 0.0169 (25P.: 0.0103, 75P.: 0.0225), Median T-CM (a.u.) = 0.0247 (25P.: 0.0179, 75P.: 0.0316), *p*=0.0149]. The texture of WGA staining exhibited decreased contrast (Fig. 3E). These differences in texture could—in part—be found using histogram-derived descriptors, such as kurtosis of extracellular staining (Fig. 3F).

In contrast to nuclear texture, nuclear orientation did not differ between the two subgroups, quantified by the OOP [Median SHAM (a.u.) = 0.0564 (25P.: 0.0159, 75P.: 0.0954), Median T-CM (a.u.) = 0.0766 (25P.: 0.0394, 75P.: 0.1027), *p*=0.3948] or OCP [Median SHAM (a.u.) = 0.8193 (25P.: 0.7501, 75P.: 0.8523), Median T-CM (a.u.) = 0.8017 (25P.: 0.7386, 75P.: 0.8468), *p*=0.4930]. However, orientation analysis using the rotation vector revealed differences between the two subgroups [Median SHAM (a.u.) = 25743 (25P.: 24226, 75P.: 30306), Median T-CM (a.u.) = 23196 (25P.: 22539, 75P.: 24002), *p*<0.0001]. Together, our MATLAB-based imaging platform allows quantification of several aspects of nuclear remodeling, such as nuclear size, texture, and orientation. The most prominent differences observed were nuclear enlargement and increased nuclear *contrast*.

### Vascular Architecture in T-CM

Given that T-CM is associated with left ventricular eccentric hypertrophy, we investigated whether vascular structure is altered in T-CM.

The total number of vessels did not differ between SHAM and T-CM animals (Fig. 3G). However, the median vessel size was increased in T-CM (Fig. 3H), while the overall size distribution remained comparable between groups (Fig. 3I). In total, vessels enlarged in T-CM.

### Interrelations of Quantitative Morphologic Descriptors

**Figure 4.**
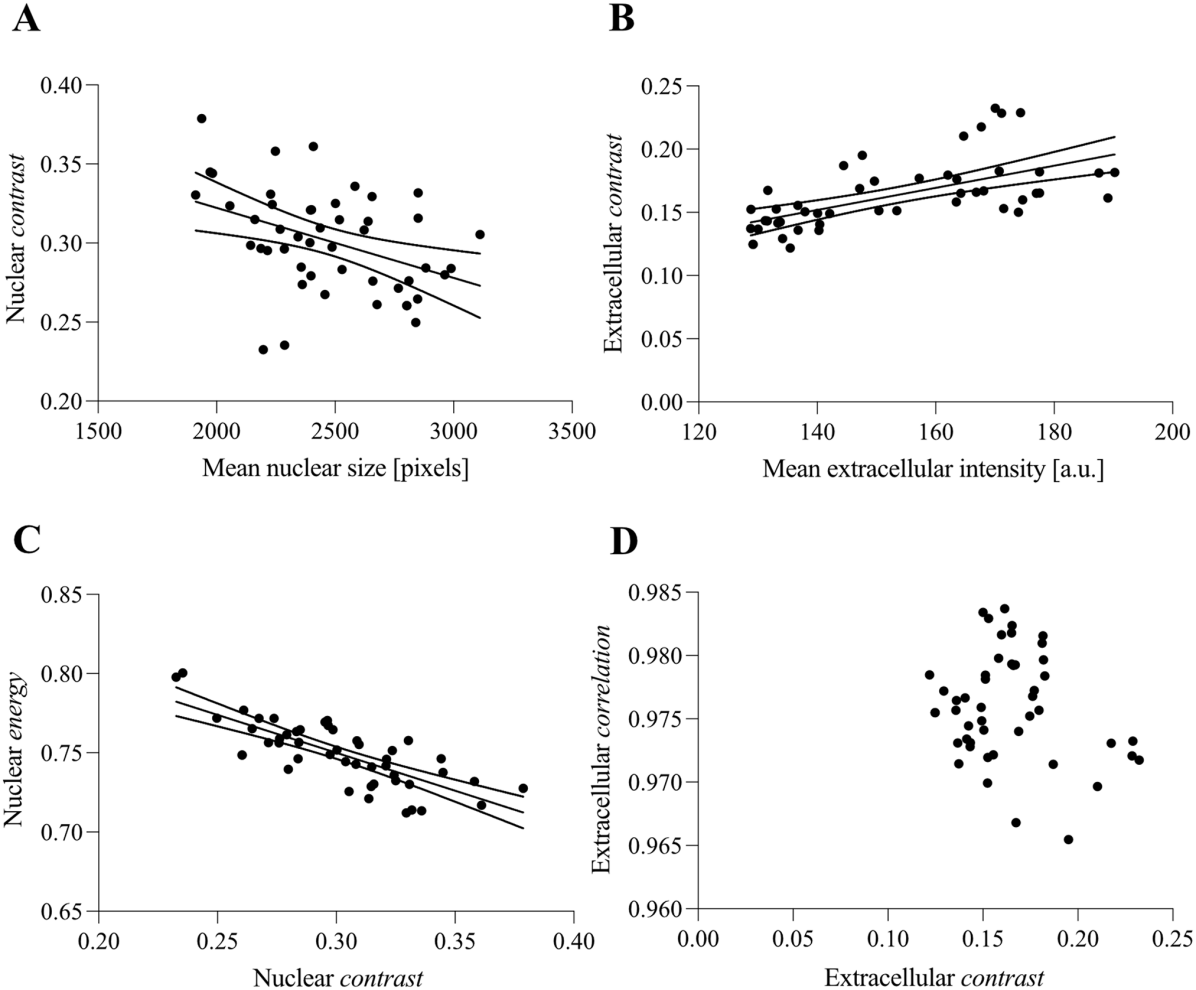
Interrelation of selected parameters. (A) Nuclear GLCM-derived contrast versus mean nuclear size expressed in number of pixels per nucleus. (B) GLCM-derived contrast from a WGA mask versus the mean WGA staining intensity. (C) GLCM-derived nuclear energy versus contrast. (D) GLCM-derived correlation of WGA mask versus contrast. Abbreviations: GLCM, gray-level co-occurrence matrix; a.u.: arbitrary unit.

To evaluate the parameters drawn from our script, it is important to cast light on selected interrelated parameters in our subset as well as methodologically related descriptors. Mean nuclear size exhibited a negative correlation with nuclear *contrast* (*r* = −0.4154, 95% CI: −0.6257 to −0.1489, *p*=0.0033, Fig. 4A). However, it is important to note that nuclear *contrast* did not correlate with DAPI staining intensity (*r* = 0.2160, 95% CI: −0.07261 to 0.4712, *p*=0.1404). The same cannot be noted for WGA staining intensity and extracellular *contrast*, which show a high correlation (*r* = 0.6133, 95% CI: 0.3989–0.7644, *p*>0.0001, Fig. 4B). Nevertheless, the extracellular *contrast* does not correlate with the number of stained pixels present (*r* = 0.1031, 95% CI: −0.1865 to 0.3762, *p*=0.4855). In addition, WGA staining intensity did not show a meaningful correlation with orientation parameters such as rotation (*r* = 0.2783, 95% CI: −0.006332 to 0.5212, *p*=0.0554). The textural descriptors have shown the greatest discriminatory effect between the two experimental groups. Due to their derivation from a GLCM, a certain interrelation is warranted. The least correlation could be observed between *contrast* and *correlation* for nuclear (*r* = −0.6691, 95% CI: −0.8010 to −0.4753, *p*<0.0001) and extracellular (*r* = −0.2075, 95% CI: −0.4643 to 0.0814, *p*=0.1570, Fig. 4D) staining. Therefore, it is noteworthy that extracellular *contrast* (see above) and extracellular *correlation* [Median SHAM = 0.9741 (25P.: 0.9716, 75P.: 0.9772), Median T-CM = 0.9781 (25P.: 0.9750, 75P.: 0.9817), *p*=0.0052] are strong discriminators of SHAM and T-CM animals even though they are not highly interrelated.

## Discussion

Tachycardia-induced remodeling differs from canonical hallmarks of ischemia-related or afterload-related cardiomyopathies.^16,22^ Despite known pathomechanistic differences, histological diagnosis of T-CM is still challenging. Tools for quantitative high-throughput analysis are scarce and limit detection of novel histological markers of T-CM as well as a clinical application.

Our study presents a comprehensive framework for the quantitative and fully automated analysis of histologic images, facilitating the stratification of cardiomyopathies based on their histologic properties. This approach enables high-throughput analysis and meticulous examination of entire hearts in experimental animal models.

We may report on nuclear remodeling in T-CM, entailing nuclear enlargement, increased nuclear contrast, and increased vessel size, potentially mirroring eccentric hypertrophy in T-CM-model^21^ and human disease.^17^ Together, the presented imaging analysis platform might help to uncover novel histological markers of T-CM in future studies.

Regarding morphometric results, it is to note that nuclear enlargement in tachycardiomyopathy is in line with known literature, where cardiomyocyte hypertrophy has been described.^16,17^ Nuclear *eccentricity* did not differ between the subgroups, suggesting omnidirectional nuclear enlargement rather than directional elongation. A decrease in nuclear *contrast* suggests that nuclear enlargement does not coincide with more heterogeneous DAPI staining. A similar heterogeneity of chromatin structure between the two groups but only dispersed over a larger nuclear area could also yield just that result.

Within our study cohort, no differences in nuclear orientation were observed between SHAM and T-CM animals. In summary, we did not observe evidence of myocardial disarray in tachycardiomyopathy, a finding that may help explain the reversibility of left ventricular systolic dysfunction in this condition.

The observed decrease in mean nuclear rotation in T-CM animals—calculated as the mean cumulative sum of edges in 180 directions—could be attributed to the smaller cellular area in SHAM animals. This higher cell border surface area per cell results in a greater number of edge pixels per direction.

As mentioned before, nuclear *contrast* negatively correlated with nuclear size. Therefore, an effect of DAPI staining dispersion over an enlarged nuclear area is a possible explanation. Further research is needed to evaluate that effect in other samples and models to estimate its relevance. Interestingly, the nuclear texture parameters *contrast* and *correlation* were—for themselves—valid discriminators between SHAM and T-CM animals. However, as they are not highly interrelated, it is possible to include them in a multivariate regression model for the discrimination of experimental groups.

Regarding nuclear segmentation, we implemented a novel method with similar accuracy to other conventional methods.^23,24^ We experienced wider applicability of nuclear segmentation compared with the implementation of watershed algorithms as our approach yields a more robust segmentation confronted with noise or disrupted nuclear borders. In comparison to nuclear segmentation using artificial intelligence, it is possible that this segmentation algorithm underperforms. Given our small sample size and the desire to use the platform in various studies with differing staining protocols and sample quality, we did not attempt to implement a segmentation algorithm using machine learning given the acceptable results for nuclear segmentation.

Although nuclear texture is often considered during pathologic evaluation, we are not aware of an automated quantitative implementation in cardiomyopathies. For texture descriptors, we used Haralick’s GLCM-derived descriptors, for they are easily computable, widely known, and have proven themselves relevant in other modalities.^25,26^ Furthermore, we implemented and established three quantitative orientation parameters for the evaluation of nuclear alignment. Future studies performed in other cardiomyopathies will show whether more can be learnt on myocardial histology based on these quantitative parameters.

## Limitations of the Study

The application of conventional discriminators, such as distance transform matrices, for identifying cardiomyocyte nuclei may lead to misclassification. Nuclei located at the sarcoplasmic borders or adjacent to vessels risk erroneous exclusion. Nevertheless, in our sample, the segmentation algorithms performed well within our subset.

The novel descriptors regarding texture and orientation warrant future evaluation in other cardiomyopathies to evaluate their discriminatory power.

## Conclusion

Our study presents a robust automated image analysis platform for quantifying histological traits in myocardial specimens. Through a multiparametric analysis workflow, the platform effectively differentiates between T-CM and healthy controls based on nuclear texture and vascular architecture. Importantly, no evidence of myocyte disarray was detected in T-CM. This platform offers new opportunities for identifying histopathological differences across cardiomyopathies.

## Appendix

**Appendix Table 1.**

Nuclear GLCM-derived texture parameters.	Median SHAM	Median T-CM	*p*
Nuclear contrast	0.3150	0.2792	0.0003
Nuclear homogeneity	0.9944	0.9950	0.0003
Nuclear energy	0.8542	0.8403	<0.0001
Nuclear correlation	0.9743	0.9757	0.0005

Abbreviations: T-CM, tachycardiomyopathy; GLCM, gray-level co-occurrence matrix.
